# Evaluating Abstract Art: Relation between Term Usage, Subjective Ratings, Image Properties and Personality Traits

**DOI:** 10.3389/fpsyg.2016.00973

**Published:** 2016-06-28

**Authors:** Nathalie Lyssenko, Christoph Redies, Gregor U. Hayn-Leichsenring

**Affiliations:** Experimental Aesthetics Group, Institute of Anatomy I, University of Jena School of Medicine, Jena University HospitalJena, Germany

**Keywords:** experimental aesthetics, descriptive evaluation, Big Five Inventory, preferences, terms

## Abstract

One of the major challenges in experimental aesthetics is the uncertainty of the terminology used in experiments. In this study, we recorded terms that are spontaneously used by participants to describe abstract artworks and studied their relation to the second-order statistical image properties of the same artworks (Experiment 1). We found that the usage frequency of some structure-describing terms correlates with statistical image properties, such as PHOG Self-Similarity, Anisotropy and Complexity. Additionally, emotion-associated terms correlate with measured color values. Next, based on the most frequently used terms, we created five different rating scales (Experiment 2) and obtained ratings of participants for the abstract paintings on these scales. We found significant correlations between descriptive score ratings (e.g., between *structure* and *subjective complexity*), between evaluative and descriptive score ratings (e.g., between *preference* and *subjective complexity*/*interest*) and between descriptive score ratings and statistical image properties (e.g., between *interest* and PHOG Self-Similarity, Complexity and Anisotropy). Additionally, we determined the participants’ personality traits as described in the ‘Big Five Inventory’ (Goldberg, 1990; Rammstedt and John, 2005) and correlated them with the ratings and preferences of individual participants. Participants with higher scores for Neuroticism showed preferences for objectively more complex images, as well as a different notion of the term *complex* when compared with participants with lower scores for Neuroticism. In conclusion, this study demonstrates an association between objectively measured image properties and the subjective terms that participants use to describe or evaluate abstract artworks. Moreover, our results suggest that the description of abstract artworks, their evaluation and the preference of participants for their low-level statistical properties are linked to personality traits.

## Introduction

In the expanding field of experimental aesthetics, the lack of a specific and well-defined terminology to describe artworks is still a major obstacle, especially in psychological studies, in which participants are asked to describe the aesthetic quality of artworks. Part of the terminological uncertainty stems from the fact that aesthetic experience represents a subjectively driven interaction between an artwork and the observer (Locher, 2011). As a consequence, aesthetic experience is highly variable between individuals and depends on a multitude of contextual and cultural factors (Leder et al., 2004).

Leder et al. (2004) introduced a widely recognized psychological model of aesthetic experience. Focusing on the effect of aesthetic experience, their model describes two aspects: (1) the aesthetic judgment, which reflects the cognitive processing of the stimulus and (2) the aesthetic emotion, which represents the affective part of the experience. Leder et al. (2004) describe aesthetic emotion as a by-product of a consecutively organized aesthetic evaluation, which includes a perceptual analysis and the explicit classification of an art object. The model also takes into account previous experiences of the beholder, amongst other factors. More recently, Redies (2015) proposed that cognitive processing of aesthetic experience and perceptual processing, which depends on image attributes [second-order image properties; see Graham and Redies (2010), take place in parallel and interact]. In his model, the cognitive processing of artworks is subject to individual preferences while the perceptual processing is more universal amongst humans.

There are different approaches to explore individual differences in the processing of artworks. Free image description is a procedure that has been rarely applied in the field of experimental aesthetics, but the few studies using this method have yielded notable results. For example, in an eye movement study, Locher et al. (2008) used free image description as a method to investigate the processing of aesthetic stimuli; they asked participants to describe eight artworks of different styles in two experiments: (1) written image description after a gist (100 ms) presentation of each image, and (2) verbal description after stimulus presentation without a time limit. The authors classified the subjects’ descriptions from both experiments into six categories of terms, which referred to the content, style, beauty, expressiveness, realism and evoked emotion of the images. Results revealed that a global impression of an artwork is formed in the beholder already after gist presentation. Moreover, initial verbalization is completed within the first 7 s. In a study on aesthetic experience and emotional content, Markovic (2010) asked participants to rate 24 paintings on the basis of a given list of 31 aesthetic adjectives (fascinating, profound, etc.) and affective adjectives (sad, lovely, etc.). The author did not find a significant correlation between aesthetic experience and affective content.

In order to assist the effective communication about artworks in experimental aesthetics research, there has been an effort to develop a universal and effective language of aesthetics for the visual domain. For example, Augustin et al. (2012) asked participants to list specific aesthetic terms for eight object categories, such as visual art, landscapes, buildings and patterns. For the category of visual art, ‘beautiful,’ ‘ugly,’ ‘colorful,’ and ‘abstract’ were the terms mentioned most frequently. ‘Beautiful’ and ‘ugly’ were general aesthetic terms applied to all categories. In another study, Markovic and Radonjic (2008) used two different approaches to gain terms for rating scales. Analogous to Augustin et al. (2012), they asked one group of participants to come up with the terms without observing stimuli. Another group of participants were asked to freely describe eight images of paintings from different styles and epochs. Then, they created rating scales from the mentioned terms from both groups in order to evaluate mostly representational art paintings. They found several correlations between implicit and explicit features.

Belke et al. (2006) demonstrated a relation between information about style in abstract paintings and the degree, to which beholders liked the artworks. Additionally, several other subjective factors can affect the observer’s aesthetic experience when viewing abstract artworks; for example, expertise in arts influences the liking of abstract art (Aluja et al., 2003; Silvia, 2006; Leder et al., 2013). For research on image description, abstract paintings are particularly useful. They have the advantage that they lack objective and figural content and thus allow for a large spectrum of possible interpretations and word usage by the participants. Redies et al. (2015) showed that there are groups of participants that have different notions of terms. In their study on abstract stimuli, some of the participants connected the term ‘aesthetic’ with the term ‘harmonious,’ while others connected it with the term ‘interesting.’ However, the authors did not investigate whether these groups of participants shared other similarities.

As a result of recent research in experimental aesthetics, specific statistical image properties (SIPs) have been associated with visual artworks. Specifically, second-order (global) image properties have been analyzed in artworks by modern computational methods (Hoenig, 2005; Graham and Redies, 2010; Mather, 2014; Redies et al., 2015). In a recent study, Mallon et al. (2014) showed that the preferences of seven subgroups of participants for abstract artworks differed systematically. They demonstrated that subjective evaluations of beauty within particular groups of participants correlated with image properties, such as PHOG Self-Similarity, Complexity and various color measures. Again, this previous study did not characterize the groups of participants in more detail.

In the present study, we therefore asked whether participants, who have similar notions of aesthetic terms and/or share preferences for artworks with certain SIPs, also share other commonalities, such as particular personality traits. Among the approaches to assess personality, the five-factor theory for personality description is a well-established model in psychological research (McCrae and Costa, 1987). The model involves the traits Neuroticism, Extraversion, Conscientiousness, Agreeableness and Openness to Experience. Previous studies based on this model showed relations of the personality of the beholder and the preference for particular art styles. For example, Furnham and Walker (2001) had participants evaluate abstract, pop and representational artworks and found a positive correlation of Neuroticism with preference for abstract and pop art, and of Conscientiousness with preference for representational paintings. Openness to Experience was associated with a higher liking of all three art categories. Other studies showed similar results. In particular, open-minded participants exhibited a higher preference for abstract art than other participants (Feist and Brady, 2004; Chamorro-Premuzic et al., 2009) and individuals with high values in Agreeableness prefer representational art over abstract art (Furnham and Avison, 1997). Furthermore, preference for abstract art is correlated with high scores in sensation seeking questionnaires (Rawlings and Bastian, 2002). As Aluja et al. (2003) pointed out; sensation seeking is positively correlated with the traits Openness and Extraversion from the Big Five Inventory (Goldberg, 1990). In conclusion, previous research indicates a preference for abstract art by open-minded and extraverted people. However, to the best of our knowledge, there has been no study that relates personality traits to the description and preference for particular artworks by individual observers. Hence, to extend the findings of previous studies, we investigated in how far personality traits affect the subjective impression that abstract paintings elicit in the beholder. To this aim, we asked participants to freely describe abstract artworks. We then obtained subjective ratings on descriptive scales and analyzed their relation to objective statistical image properties (SIPs). A detailed account of the included SIPs can be found in Section “Statistical Image Properties.”

The present study is thus an effort to define the verbal terms that are associated with aesthetic experience and to elucidate some of the perceptual features that underlie differential term usage by participants with varying personality traits. We focus on two main research questions: (1) How varied are descriptions of abstract artworks? (2) Can the personality traits of the observers be associated with their term usage for abstract artworks and with a preference for images with certain SIPs?

## Materials and Methods

### Ethics Statement

The study was approved by the ethics committee of the Universitätsklinikum Jena. The participants stated their consent by signing a detailed consent form. No vulnerable populations were involved.

### Experiment 1 – Verbal Description of Images

#### Participants

19 participants (19–37 years old; *Mean* = 22.8 years; six male), mostly medical students, participated in the experiment. All participants were native German speakers and none reported expertise in visual arts. One participant of the original sample of 20 participants was excluded because of technical problems with data collecting.

#### Stimuli

**FIGURE 1:**
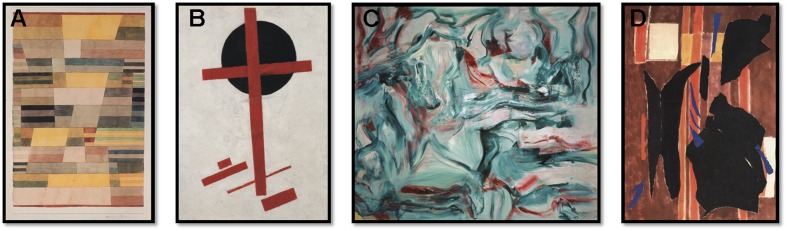
**Examples for test images.** **(A)** Monument im Fruchtland, Paul Klee, 1929; **(B)** Mystic Suprematism (red cross on black circle), Kazimir Malevich, 1920–1922; **(C)** Untitled VIII, Willem de Kooning, 1980(c) The Willem de Kooning Foundation, New York/VG Bild-Kunst, Bonn, 2016; **(D)** Stretched Yellow, Lee Krasner, 1955(c) Pollock-Krasner Foundation/VG Bild-Kunst, Bonn, 2016.

For the experiment, 79 images were chosen from the collection of 150 images of abstract artworks that was compiled by Mallon et al. (2014). We restricted image choice to abstract paintings of the 20th and 21st century, because of their relatively lack of semantic content (see **Figure 1** for example images). There was a maximum of two images per artist to minimize the influence of individual preferences or the recognition of single artists on the result. Otherwise, the images were selected randomly from the database. All of the images were high-quality scans from different art books. The images were reduced to a size of 1024 pixels on the longest side and were presented at a maximum size of 165 mm (9.5° of visual angle) on the screen. They were presented on a calibrated screen (EIZO ColorEdge CG241W) at a constant viewing distance of 100 cm, ensured by a chin rest.

#### Statistical Image Properties

By means of a previously established MATLAB algorithm (Amirshahi et al., 2012), we calculated the following SIPs for every single image from the dataset:

(1) PHOG Self-Similarity. Here, we calculated Self-Similarity using the Pyramid Histogram of Orientation Gradients (PHOG) method that was introduced by Bosch et al. (2007). The algorithm is based a comparison of histograms of oriented gradients (HOGs) from the entire image with HOGs from equal subparts of the image. For a detailed description of the procedure, see the Appendix in Braun et al. (2014). Self-Similarity, a concept closely related to scale-invariance and fractality, implies that an object has a structure similar to its parts. Museum paintings exhibit a relatively high degree of Self-Similarity compared to other image categories (Amirshahi et al., 2012, 2013; Redies et al., 2012).
(2) HOG Complexity. Berlyne (1974) postulated that an intermediate complexity of stimuli leads to a higher aesthetic appeal than low or high complexity (Nadal, 2007). Recently, several studies confirmed the role of complexity in beauty perception (Jacobsen and Hofel, 2002; Rigau et al., 2008; Forsythe et al., 2011). We defined HOG Complexity as the sum of the strengths of the oriented gradients in the image as described by Braun et al. (2014).
(3) Anisotropy. Anisotropy is a measure for the distribution of orientation of gradients within a particular image. Low Anisotropy implies that the strength of luminance gradients is uniformly distributed across all orientations; high values indicate that one or a few orientations are represented more strongly than others in the orientation spectrum. Previous studies showed that colored artworks show a relatively low degree of Anisotropy compared to other categories of images (Redies et al., 2012). We calculated Anisotropy as described by in Braun et al. (2014).
(4) Aspect Ratio. Although there is no evidence for an overall preference of a certain format of paintings (McManus, 1980; Russell, 2000), we used this measure to investigate whether it is correlated with the subjective description of images and whether certain groups of participants preferred a certain aspect ratio over others in abstract artworks. The measure was obtained by dividing image height by image width.
(5) Color measures. In addition to second-order image statistics, we calculated the three color measures of the HSV color space (Color Hue, Color Saturation and Color Value), which have been used in aesthetic quality assessment of images previously (Datta et al., 2006). Previous studies described a link between color and emotion (Ou et al., 2004a). We calculated the color measures by means of a MATLAB algorithm. The HSV values were computed pixel-by-pixel. For each of the three color measures, the mean across all pixels was taken as the final value.

Throughout the text, the above image properties are capitalized.

#### Procedure

The participants were tested separately in front of a screen in a shaded room (windows covered by blinds). First, the instructions for the experiment were displayed on the screen. The participants started the experiment with a mouse click. Then, a fixation cross appeared on the screen for between 300 and 800 ms followed by the first image. Each image was displayed for 30 s. The order of the images was random. After every image, there was a black screen for 3 s. During presentation, participants were asked to describe the image by using German adjectives. The adjectives used to describe the images were translated into English and are italicized in the text; the original German terms are parenthesized. We decided to restrict the word pool to adjectives, a category of terms that is frequently used to characterize (aesthetic) objects (mostly in conjunction with rating scales; Cupchik and Gebotys, 1990; Markovic, 2010). The present study can thus be compared to previous studies. The experimenter noted the verbalized terms on another computer. The experimenter sat in a different corner of the room with the back turned to the participant to minimize any interference (e.g., by eye contact).

### Experiment 2 – Rating Scores

#### Participants and Stimuli

Forty-two participants (19–31 years old, *Mean* = 23.5 years; 14 males), who had not participated in Experiment 1, took part in the experiment. As in Experiment 1, participants were medical students and native German speakers with no reported expertise in the arts. Stimuli were the 150 images of abstract art collected by Mallon et al. (2014). The setting was analogous to Experiment 1.

#### Procedure

Participants rated the images on five subjective ratings scales. The terms used for the scales were 8 of the top 10 most commonly used terms (or term groups) from Experiment 1. We built contrast term couples to create the scales. The term couples of the scales were *structured/unstructured*, *complex/simple, pleasant/sad*, and *interesting/uninteresting*. The scales are called *structure* scale*, subjective complexity* scale*, valence* scale and *interest* scale, respectively. Although the terms *pleasant* and *sad* (*valence* scale) are no true antagonists, we used them based on the assumption that participants associate these terms with positive or negative emotion, respectively. The term *colorful* had a high number of mentions in Experiment 1. Nevertheless, we excluded it because we did not focus on color descriptions of the images. Furthermore, *beautiful* was excluded because of its ambiguity. Instead, we used a scale for *preference* (ranging from *do not like* (gefällt mir nicht) to *like* (gefällt mir)). The range of each scale was from 0 (first term from the scale descriptions above) to 1 (second term) in 100 steps that were not visible to the participants. Only the ends and the middle of the scale were indicated with vertical lines. For the analysis, we converted the scales. Thus, high values in the *structure* scale represent images subjectively rated as *structured* and so on. The participants rated all of the 150 images for a total of five times on the five different scales. The order of the scales and of the images was randomized. Each trial started with a fixation cross that was presented on the screen for between 300 and 800 ms. Afterward, the image was shown for 3 s, followed by a black screen with the rating scale. The participants provided their ratings for the particular images on the respective scale by a mouse click with no time limit for answering. Then, the next trial was initiated.

Before or after the experiment, participants were asked to complete the BFI-K, the short version of the Big Five Inventory (Rammstedt and John, 2005). Compared to the full version, the BFI-K has acceptable reliability coefficients and validity (Rammstedt and John, 2005). The BFI-K measures the personality traits Neuroticism, Extraversion, Conscientiousness, Agreeableness and Openness. Throughout the text, the BFI-K personality traits are capitalized.

## Results

### Results Experiment 1

Overall, participants used 1447 different terms (4447 terms in total). Per image, participants used 47.92 different terms on average. Each person used a *Mean* of 135.31 different terms for all images (range of 68–253 different terms). The most frequently used term was *interesting* (interessant, 77 mentions).

To reduce the number of terms, we pooled synonymous (and similar) terms, like *disordered*, *unstructured* and *chaotic* (see **Supplementary Table S1** for translation of the used terms and a description of the pooling process). In the next step, we counted the most frequently used terms or term pools. If a participant used more than one term from the same word pool to describe a particular image, it was counted as one mention only. The 10 most commonly used terms (after pooling) were *dark* (156 mentions), *unstructured* (151), *structured* (132), *interesting* (94), *beautiful* (81), *boring* (71), *colorful* (70), *simple* (68), *pleasant* (63), and *sad* (58). Most of these terms were used in Experiment 2.

For the 10 most frequently used terms, we correlated the frequency of term usage with the SIPs. For the 79 abstract images used in Experiment 1, we found significant correlations between term usage frequency and the SIPs. PHOG Self-Similarity correlated positively with *unstructured* (*r* = 0.417; *p* < 0.001) and negatively with *structured* (*r* = -0.453; *p* < 0.001) and *simple* (*r* = -0.472; *p* < 0.001). Correlations were found also for HOG Complexity with *unstructured* (*r* = 0.659; *p* < 0.001), *structured* (*r* = -0.277; *p* < 0.05) and *simple* (*r* = -0.524; *p* < 0.001) as well as *boring* (*r* = -0.303; *p* < 0.001) and *colorful* (*r* = 0.240; *p* < 0.05). Furthermore, Anisotropy correlated with *structured* (*r* = 0.444; *p* < 0.001). There were significant positive correlations of Color Value with *pleasant* (*r* = 0.344; *p* < 0.05) and negative correlations with *sad* (*r* = -0.526; *p* < 0.001) and *dark* (*r* = -0.678; *p* < 0.001; see **Table 1** for a detailed analysis).

**Table 1:** Pearson’s *r* for correlations betwen usage frequency of descriptive terms and statistical image properties (^∗^*p* < 0.05; ^∗∗^*p* < 0.001).

Term	PHOG self-similarity	HOG complexity	Anisotropy	Aspect ratio	Color Hue	Color saturation	Color value
*Dark*	0.031	-0.164	-0.134	-0.072	0.198	-0.194	-0.678ˆ**
*Unstructured*	0.417ˆ**	0.659ˆ**	-0.232ˆ*	-0.138	-0.123	-0.055	0.051
*Structured*	-0.453ˆ**	-0.277ˆ*	0.444ˆ**	0.099	0.007	-0.188	0.140
*Interesting*	-0.123	0.072	-0.056	0.193	-0.057	0.086	-0.047
*Beautiful*	0.069	0.059	-0.153	-0.001	-0.135	0.184	-0.047
*Colorful*	0.155	0.240ˆ*	0.091	-0.028	-0.131	0.179	-0.036
*Boring*	-0.135	-0.303ˆ**	0.071	-0.144	0.108	-0.143	0.125
*Simple*	-0.472ˆ**	-0.524ˆ**	0.177	0.005	0.148	-0.080	0.099
*Happy*	-0.026	-0.033	0.140	-0.002	-0.146	0.177	0.344ˆ**
*Sad*	0.106	-0.070	-0.238ˆ*	-0.176	0.221	-0.161	-0.526ˆ**
*Cold*	0.195	0.107	-0.109	-0.017	0.432ˆ**	-0.187	-0.068
*Warm*	0.047	-0.244ˆ*	-0.071	0.124	-0.373ˆ**	0.557ˆ**	-0.011


*Displayed are the 12 most frequently mentioned terms (after pooling).*

### Results Experiment 2

We calculated the mean rating score for every image for each scale and then focused on two questions: (1) Do the different rating scores correlate with each other and/or SIPs? (2) Are there interactions with the BFI-K scores?

The evaluation on the *preference* scale was positively correlated with the evaluation on the *subjective complexity* scale (*r* = 0.317; *p* < 0.001), with the evaluation of the *valence* scale (*r* = 0.380; *p* < 0.001) and with the evaluation on the *interest* scale (*r* = 0.376; *p* < 0.05). Furthermore, evaluations on the *structure* scale had a negative correlation with evaluations on the *subjective complexity* scale (*r* = -0.488; *p* < 0.001) and a positive correlation with evaluation on the *interest* scale (*r* = 0.405; *p* < 0.001). Last but not least, the *subjective complexity* and the *interest* scale correlated positively (*r* = 0.503; *p* < 0.001). See **Table 2** for complete data. Results show that *valence* did not correlate with any descriptive evaluations. To summarize, participants preferred paintings that were *more subjectively complex*, *interesting* and *pleasant*.

**Table 2:** Pearson’s *r* for correlations between ratings on the descriptive scales from Experiment 2.

	Interest	Subjective complexity	Structure	Valence
Preference	0.376^∗∗^	0.317^∗∗^	0.03	0.380^∗∗^
Interest		0.503^∗∗^	0.405^∗∗^	0.054
Subjective complexity			-0.488^∗∗^	0.058
Structure				-0.001


*^∗∗^*p* < 0.001.*

**FIGURE 2:**
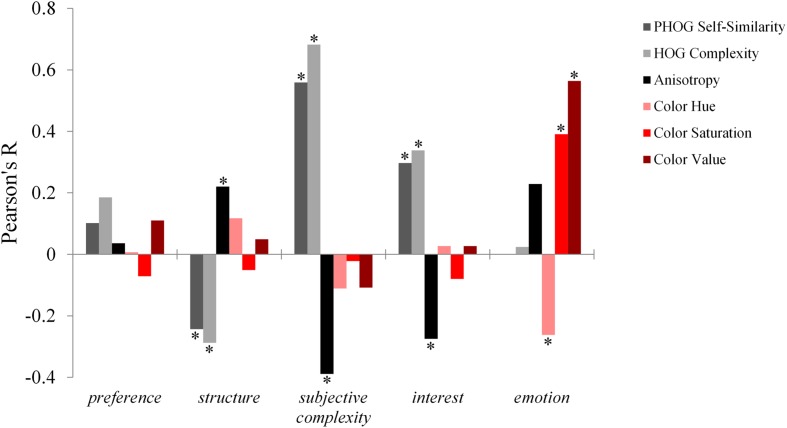
**Correlations of ratings on subjective scales with SIPs.** Second-order image properties are displayed in greyscale, whereas color measures are displayed in shades of red. ^∗^*p* < 0.001.

Next, we investigated whether subjective ratings are linked to SIPs. The evaluation on the *structure* scale was positively correlated with Anisotropy (*r* = 0.220; *p* < 0.001) and negatively with PHOG Self-Similarity (*r* = -0.243; *p* < 0.001) and with HOG Complexity (*r* = -0.288; *p* < 0.001). Evaluations on *subjective complexity* correlated positively with PHOG Self-Similarity (*r* = 0.559; *p* < 0.001) and with HOG Complexity (*r* = 0.682; *p* < 0.001) and negatively with Anisotropy (*r* = -0.389; *p* < 0.001). A rather similar pattern (but with lower correlations) was found for evaluations on *interest*, which also correlated positively with PHOG Self-Similarity (*r* = 0.297; *p* < 0.001) and with HOG Complexity (*r* = 0.388; *p* < 0.001) and negatively with Anisotropy (*r* = -0.275; *p* < 0.001). The evaluation on *valence* did not show any significant correlations with second-order SIPs. Instead, we found correlations with color measures, namely with Color Hue (*r* = -0.262; *p* < 0.001) with Color Saturation (*r* = 0.391; *p* < 0.001), as well as with HOG Complexity (*r* = 0.564; *p* < 0.001). In summary, the subjective *pleasantness* was influenced by the coloring of the images, while second-order SIPs are linked to evaluations on the other descriptive scales. Interestingly, the mere liking of images is not correlated with any the objectively measured values (see **Figure 2** for complete results).

In order to investigate differences according to the BFI-K personality of the participants, we calculated (1) the correlations of the subjective rating scales among each other, as well as (2) the correlations of the subjective rating scales with the SIPs for each participant individually. Next, a stepwise multiple linear regression was run to investigate whether these correlations are correlated with BFI-K personality traits. The personality trait was the dependent variable while correlations of subjective rating scales with each other and with the SIPs for each participant were considered independent variables in the analysis. Results were controlled with a *post hoc* Holm–Bonferroni method. Two of the correlations of the subjective rating scales predicted Extraversion [*R^2^* = 0.339; *F*(2,39) = 10.014; *p* < 0.001]. These were the correlation of the *structure* scale with the *subjective complexity* scale (β = 0.486; *p* < 0.001), as well as the correlation of the *structure* scale with the *valence* scale (β = 0.441; *p* < 0.01). Therefore, participants with higher scores for Extraversion seem to have a different notion of the term structured than participants with lower scores for Extraversion, who relate the term structured to *subjectively complex* and *pleasant* images.

Additionally, Neuroticism [*R^2^* = 0.695; *F*(7,34) = 11.043; *p* < 0.001] was predicted by the correlations of the *preference* scale with HOG Complexity (β = 0.504; *p* < 0.001) and the correlation of the *subjective complexity* scale with three SIPs, namely PHOG Self-Similarity (β = 0.438; *p* < 0.05), Color Hue (β = 0.434; *p* < 0.01) and Anisotropy (β = 0.497; *p* < 0.01), as well as with the *structure* scale (β = 0.510; *p* < 0.001). Additionally, the correlation of the Aspect Ratio with the *interest* scale (β = 0.462; *p* < 0.001) as well as the *valence* scale (β = 0.242; *p* < 0.05) were also predictive. These results indicate, for instance, that participants with higher scores for Neuroticism prefer more objectively complex images and, additionally, they have a different notion of the term *complex* as compared to participants with lower scores for Neuroticism.

Openness [*R*^2^ = 0.321; *F*(2,39) = 9.233; *p* < 0.001] was predicted by the correlation of the *preference* scale with Aspect Ratio (β = -0.558; *p* < 0.001) and the correlation of the *interest* scale with HOG Complexity (β = -0.359; *p* < 0.05). Therefore, participants with higher scores for Openness prefer landscape-orientated abstract artworks, while participants with lower scores for Openness find objectively complex images more *interesting*.

There was no effect for the other personality traits (Conscien tiousness and Agreeableness).

## Discussion

### Description of Abstract Artworks

In this study, participants freely described abstract artworks in a highly versatile way although the word choice was restricted to adjectives (Experiment 1). Overall, 19 participants used 1447 different terms to describe 79 images of abstract artworks. The spectrum of descriptions ranged from style- and composition-related terms to emotional expressions and preference evaluations. The observed individual differences in usage of diverse terms were large. Nevertheless, participants often used similar terms to describe the same stimulus, reflecting a relative constancy in term usage for the description of particular images.

In a previous study, Augustin et al. (2012) showed that ‘beautiful,’ ‘ugly,’ and ‘abstract’ were the terms mentioned most frequently when people were asked which words they associated with visual arts. In contrast to their study, in which participants did not actually observe any artworks, our study provides evidence that, when confronted with images of abstract art during the experiment, participants frequently use other terms, namely *dark*, *unstructured*, *structured*, and *interesting*. We hypothesize that the difference to the results of Augustin stems from the fact, that when participants are confronted with actual stimuli, they have a concrete visual experience, which drives a more sophisticated word choice.

### Evaluation of Abstract Artworks

The rating scores correlated between the different scales, which had been created in accordance with the results of Experiment 1. For example, the more participants found the artwork *subjectively complex*, the more they also found it *interesting* and liked it (Experiment 2), with a moderate correlation (Pearson’s *r* ≈0.4). This outcome confirms similar results from previous studies by Berlyne (1970) and Silvia (2005) for non-representational grayscale images. Furthermore, subjectively more *interesting* abstract images were preferred overall. In a study on portrait images, Leder et al. (2013) obtained similar results for ‘interestingness’ and ‘likeability’ ratings. In conclusion, *liking*, *interest* and *subjective complexity* are related terms in the description of abstract art.

Markovic (2010) focused on aesthetic and affective descriptive adjectives and found no significant interaction between those groups. Here, we found differences between different personality groups in the correlations of their usage of descriptive and evaluative terms. Participants with higher scores for Extraversion related *structure* to *subjective complexity* and *valence*. Thus, we hypothesize that these participants possessed a different concept of *structure* and/or *subjective complexity* than participants with lower scores for Extraversion. Additionally, the subjective impression of a more *structured* image is associated with a more positive emotional evaluation of it (*valence* scale). Therefore, we conclude that the subjective interpretation of descriptive aesthetic terms varies between people with different personality traits. Our data extend the findings by Markovic (2010) by showing that aesthetic and descriptive evaluations are related, especially when personality traits of the participants are considered. Thus, our results suggest that the personality of the participants should be accounted for in empirical aesthetics research.

Previously, Redies et al. (2015) reported that PHOG Self-Similarity is inversely correlated and HOG Complexity is positively correlated with *interest* ratings of abstract stimuli. Our data confirms the finding on HOG Complexity while it contradicts the finding for PHOG Self-Similarity. This contradiction possibly originates from the different types of stimuli and different mean values of PHOG Self-Similarity (Redies et al. (2015): *Mean*: ∼0.7; our study: *Mean* = 0.55).

We also found correlations for the usage of particular terms with SIPs of the abstract art images. In particular, PHOG Self-Similarity and Anisotropy values of the images correlated with the number of mentions of the terms *structured* and *boring*, amongst others (Experiment 1). Results were confirmed by the evaluation based on fixed rating scales (Experiment 2). Together, these results indicate that the verbal description of images correlates with specific SIPs and is therefore far from arbitrary with respect to structural image features that can be processed at low levels of the visual system.

Next, we analyzed the correlations between the ratings for the subjective scales and the SIPs. We found correlations of the *valence* ratings with color measures (Color Hue, Color Saturation and Color Value). Bright, highly saturated and red/yellowish abstract artworks are linked to a positive *valence* (*pleasant*). A similar association of emotions and specific colors had been described in previous research (Ou et al., 2004a,b; Palmer and Schloss, 2010).

Three of the rating scales (*structure*, *subjective complexity* and *interest*) showed no correlations with color measures, but with non-color SIPs. On the one hand, s*tructure* and *subjective complexity* are descriptive terms and ratings on these scales characterize composition-related image properties. Therefore, correlations with the SIPs are not surprising. On the other hand, the term *interesting* is evaluative and generally associated with aesthetic appeal. It has been shown previously that self-similar abstract images are rated as less *interesting* (see Figure 8H in Redies et al., 2015). This result was confirmed in the present study. Furthermore, participants also found more objectively complex and more isotropic images more *interesting*.

For Neuroticism, we found a significantly different evaluation on the *subjective complexity* scale than for participants with lower scores for Neuroticism. This is not only established in the relation of *subjective complexity* to another subjective term (*structure*), but also in evaluation of objectively more self-similar and less isotropic images as more *complex*. We therefore conclude that participants with higher scores for Neuroticism have a different notion of the term *complex* than participants with lower scores for Neuroticism.

### Preferences for Abstract Artworks

Previous studies showed that people with different personality traits exhibit a preference for particular art styles. Specifically, participants with higher scores for Neuroticism, Extraversion and Openness like abstract artworks more than other artistic styles (Furnham and Avison, 1997; Furnham and Walker, 2001; Rawlings and Bastian, 2002). Here, we provide evidence that even for one particular art style (i.e., abstract art), aesthetic preferences depend on individual personality traits. The present study is a follow-up to a study on image statistics by Mallon et al. (2014), who showed that subgroups of participants prefer images with different SIPs. Here, we extend these previous findings and show that high values in Neuroticism are linked to a preference for objectively complex images, while high values in Openness can be associated with a preference for a portrait orientation of images. Güclütürk et al. (2016) described that two groups of participants differed in their liking of digital images with varying complexity. One group of participants showed increasingly lower liking rates for increasingly more complex images while another group showed the opposite pattern of preference. Here, we extend these findings by showing that, in addition to their general preference for abstract artworks (Furnham and Walker, 2001), participants with higher scores for Neuroticism also prefer more objectively complex abstract artworks as compared to participants with lower scores for Neuroticism.

### Limitations

One limitation of the present study is the mode of presentation of the artworks. By presenting mere images of abstract artworks on a computer screen, it is hardly possible to evoke an aesthetic state of mind. Therefore, our study focuses on preferences rather than on beauty judgments or aesthetic experiences. Another problem is the choice of participants, who were mostly young German students. Therefore, it is difficult to generalize our results to the general population, in particular the findings related to the BFI-K classification. Future studies are needed to assess whether our findings can be generalized.

## Conclusion

In summary, individual personality traits and objectively measured image properties play a role in (1) the evaluation on descriptive scales of abstract images and (2) the individual preferences for them. Potentially, such individual differences in term usage complicate aesthetic research. Augustin et al. (2012) suggested that ‘beauty’ is a good choice of term if one wants to investigate aesthetic impressions with a single-item measure. However, our results suggest that aesthetic experience is associated with a very wide range of adjectives. We therefore propose that research in experimental aesthetics should be conducted in a more versatile way by using multiple different terms (like ‘interesting,’ ‘structured,’ and ‘pleasant,’ among others). Admittedly, a clear definition of the terminology used is of utmost importance. Lacking this definition, results will not be comparable, because individual participants may use the same terms, but imply different meanings.

Our results might have implications for modeling aesthetic experience. We were able to demonstrate that descriptive term usage and preference for modern artworks are related to perceptual processing and depend on SIPs. In other words, aesthetic experience – as induced by abstract artworks – seems to depend on perceptual processing of measurable properties, such as image attributes (of the perceived object) and personality traits (of the perceiving subject). Our results might have implications for modeling aesthetic experience. We were able to demonstrate that descriptive term usage and preference for modern artworks are related to perceptual processing and depend on SIPs. In other words, aesthetic experience – as induced by abstract artworks – seems to depend on perceptual processing of measurable properties, such as image attributes (of the perceived object) and personality traits (of the perceiving subject). For example, Leder et al. (2004) proposed a more or less consecutive model, in which perceptual processing takes place at a low level, and is then followed by cognitive processing of explicit and contextual information at successively higher levels of the model. The authors state that higher processing potentially effects previous processing stages. Possibly, our findings reveal one such feedback loop because we can show that personality traits have an effect on the individual preference for physical image attributes (SIPs) in the images. The described results are also in line with other models (Chatterjee and Vartanian, 2014; Redies, 2015). Redies (2015) speculated that perceptual processing and cognitive processing take place in parallel to interact and converge on aesthetic experience at higher levels. Although not decisive, our findings favor a parallel model, in which perceptual processing and cognitive processing take place independently and both contribute to aesthetic experience

## Author Contributions

NL designed the experiment, collected data, did the statistical analyses and wrote the manuscript. CR helped designing the experiment and revised the manuscript. GH-L designed the experiment, collected data, did the statistical analyses and wrote the manuscript.

## Conflict of Interest Statement

The authors declare that the research was conducted in the absence of any commercial or financial relationships that could be construed as a potential conflict of interest.
