# Supplementary material for: Evaluating Abstract Art: Relation between Term Usage, Subjective Ratings, Image Properties and Personality Traits
Source: Front Psychol. 2016 Jun 28;7:973. doi: 10.3389/fpsyg.2016.00973 (PMC4923070; doi:10.3389/fpsyg.2016.00973)
Supplement: Supplementary file 1 [file Table_1.DOCX]

| **German terms** | **English translation** | **Pooled terms (German)** |
| --- | --- | --- |
| aggressiv | *aggressive* | wütend, zornig |
| alt | *old* | altbacken |
| aufgeregt | *agitated* | nervös |
| aussagekräftig | *meaningful* | vielsagend, ausdrucksstark, bedeutungsschwanger |
| aussagelos | *meaningless* | sinnlos, nichtssagend |
| beklemmend | *oppressive* | zerdrückend, beengend |
| beweglich | *agile* | tanzend, tänzelnd, quirlig, bewegt, dynamisch |
| böse | *mean* | grausam, gemein |
| bunt | *colorful* | farbenfroh, farbig |
| dreckig | *dirty* | matschig, erdig |
| dunkel | *dark* | düster |
| eckig | *angulary* | kantig |
| einfach | *simple* | simpel, schlicht, reduziert |
| eintönig | *monotonous* | monoton, einfarbig |
| emotional | *emotional* | gefühlvoll |
| fein | *fine* | filigran, grazil, sanft, zart, leicht |
| fleckig | *spotted* | gekleckst, klecksig, befleckt, tüpfelig, getupft |
| flüssig | *fluent* | fließend |
| frei | *free* | freiheitlich |
| freundlich | *pleasant* | heiter, nett, lieb, fröhlich, freudig |
| gegensätzlich | *oppositional* | kontrastreich |
| gerade | *straight* | geradlinig, linear, gradlinig |
| geteilt | *split* | gespalten, getrennt, zerlegt, zerrissen, zerbrochen, zerschneidend, zerteilt, abgetrennt, gesplittert |
| glühend | *fiery* | feurig |
| hässlich | *ugly* | unschön |
| hoffnungsvoll | *hopeful* | hoffnungsbringend |
| interessant | *interesting* | faszinierend, spannend, aufregend, mitreißend, aufmerksamkeitserregend |
| isoliert | *isolated* | einsam, singulär, verloren |
| kalt | *cold* | eisig, kühl, winterlich, vereist |
| klobig | *bold* | plump |
| komplex | *complex* | verstrickt, vielfältig |
| kreativ | *creative* | fantasiereich, phantasievoll |
| langweilig | *boring* | trist, uninteressant, öde |
| lebendig | *vital* | vital |
| lustig | *funny* | spaßig, witzig |
| modern | *modern* | jung, jugendlich, neu, postmodern |
| mystisch | *mystic* | märchenhaft |
| natürlich | *native* | biologisch, naturhaft, lebensnah |
| ruhig | *calm* | entspannt, beruhigend |
| rund | *round* | kreisförmig |
| schmierig | *greasy* | schleimig |
| schön | *beautiful* | toll, wunderschön, wundervoll, hübsch |
| schwebend | *pending* | freischwebend |
| sonderbar | *odd* | skurril, bizarr, seltsam, merkwürdig, verwundernd, verrückt, eigenartig |
| sonnig | *sunny* | lichtvoll |
| spitz | *pointy* | igelig, stachelig, kratzig |
| stark | *strong* | kraftvoll |
| strukturiert | *structured* | definiert, klar, geordnet, ordnend, ordentlich, angeordnet |
| tot | *dead* | tödlich |
| traurig | *sad* | melancholisch, depressiv, betrübt, bedrückend, deprimierend |
| unangenehm | *displeasing* | eklig, abstoßend |
| unruhig | *anxious* | aufwühlend |
| unstrukturiert | *unstructured* | ungeordnet, durcheinander, wüst, durcheinander, chaotisch, wirr, verworren, konfus, durcheinandergewirbelt |
| verbunden | *connected* | zusammenhaltend |
| verführerisch | *attractive* | sinnlich, anziehend, erotisch, verlockend |
| verletzend | *offending* | gewalttätig, vergewaltigend, zerstörerisch |
| verschwommen | *blurred* | verwaschen, verlaufend, verwischt, unscharf, verschmiert |
| vielseitig | *versatile* | facettenreich, abwechslungsreich, vielschichtig |
| weit | *wide* | weitreichend, weiträumig, weitblickend, weitgründig, tief |
| zentriert | *centered* | zentralisiert, zentralisierend, mittig |
| zielorientiert | *targeted* | zielstrebig, zielweisend, zielgerichtet, weisend |
| zufällig | *random* | spontan |
| zwiegespalten | *diverging* | auseinanderstrebend, auseinanderberstend |

Supplementary Table 1: Pooled terms in German. The experimenters grouped the mentioned adjectives (right hand side) under the respective individual terms (left hand side, with English translations).
